# Computing symmetrical strength of N-grams: a two pass filtering approach in automatic classification of text documents

**DOI:** 10.1186/s40064-016-2573-y

**Published:** 2016-06-30

**Authors:** Deepak Agnihotri, Kesari Verma, Priyanka Tripathi

**Affiliations:** Department of Computer Applications, National Institute of Technology Raipur, Raipur, CG India; Department of Computer Engineering and Applications, National Institute of Technical Teachers Training & Research Bhopal, Bhopal, MP India

**Keywords:** Feature selection, Text classification, Text analysis, Text mining, Information retrieval

## Abstract

The contiguous sequences of the terms (N-grams) in the documents are symmetrically distributed among different classes. The symmetrical distribution of the N-Grams raises uncertainty in the belongings of the N-Grams towards the class. In this paper, we focused on the selection of most discriminating N-Grams by reducing the effects of symmetrical distribution. In this context, a new text feature selection method named as the symmetrical strength of the N-Grams (SSNG) is proposed using a two pass filtering based feature selection (TPF) approach. Initially, in the first pass of the TPF, the SSNG method chooses various informative N-Grams from the entire extracted N-Grams of the corpus. Subsequently, in the second pass the well-known Chi Square (χ^2^) method is being used to select few most informative N-Grams. Further, to classify the documents the two standard classifiers Multinomial Naive Bayes and Linear Support Vector Machine have been applied on the ten standard text data sets. In most of the datasets, the experimental results state the performance and success rate of SSNG method using TPF approach is superior to the state-of-the-art methods viz. Mutual Information, Information Gain, Odds Ratio, Discriminating Feature Selection and χ^2^.

## Background

The increasing add up of text data on the web, necessitates efficient techniques or tools (like Text Mining) that automatically arrange text documents into known classes^1^$$^{,}$$^2^$$^{,}$$^3^ has given ascend to the field of text documents classification (Joachims 1996). The classification of text documents, based on their contents is a real challenging problem due to high dimensionality. In the Automatic Text Document Classification (ATDC) process, the relevant features play an important role. The selection of most relevant feature is an important task to reduce the dimensionality and to increase the performance of the classifiers in ATDC (Sharma and Dey 2012; Joachims 1998).

**Fig. 1:**
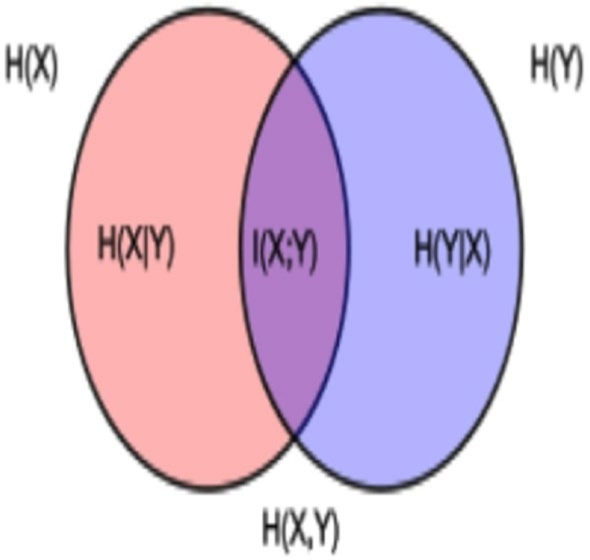
Symmetrical information of an N-Gram

In the information theory, the various information measurement methods viz. MI, IG, OR, DFS, and $\chi ^2$ are used to compute association between correlated variables X (N-Gram $NG_{i}$) and Y (class $c_{r}$). These methods are not fare enough to compute the nature of the N-Gram—common, rare or sparse along with their symmetrical uncertainty towards the classes. The symmetrical information of the N-Gram $NG_{i}\in X$ associated with class $C_{j}\in Y$ can be represented by Fig. 1. In Fig. 1, the area contained by both the circles is the joint entropy *H*(*X*, *Y*). The circle in the left (red and violet) is the individual entropy *H*(*X*), with the red being the conditional entropy *H*(*X*|*Y*). The circle on the right (blue and violet) is *H*(*Y*), with the blue being *H*(*Y*|*X*). The violet is the symmetrical information *I*(*X*; *Y*).^4^

The representation of the terms of the corpus is the base to determine the computational informativeness of the terms to classify the text documents automatically. The Bag of Words (BOW) model is the basic model to represent the terms. It is a simplified representation of terms, used in the natural language processing and information retrieval. In this model, a text (such as a sentence or a document) is represented as the bag (multi set) of its individual words, disregarding grammar and word order but keeping its multiplicity. The BOW model uses the occurring frequency of the terms as the base criteria to discriminate the terms of the class documents. The major drawback of the BOW model is that, here the order of term occurrence is not important, only the occurring frequency of the term is considered.

The N-gram language (NGL) model (Duoqian et al. 2009) has solved this problem up-to some extent by considering the order of term occurrence in the sentences of various class documents. The N-Gram is a contiguous sequence of n terms in a given text. In the NGL model, the various combinations of terms occurred together in the sentences of various documents is combined as a set. E.g., suppose we have to classify a sentence, “I do not like the story of the movie” as positive or negative? Since this document contains N-Gram “like”, by using conventional BOW model may be misclassified as positive document. In such cases, we need a combination of two or more N-Grams “not like” or “do not like” known as N-grams words.

This article investigates about the barriers in ATDC. The contiguous sequences of the terms (N-grams) in the documents are symmetrically distributed among different classes. The symmetrical distribution of the N-Grams raises uncertainty in the belongings of the N-Grams towards the class. In the symmetrical distribution, the nature of an N-Gram might be common, rare or sparse. The common N-Grams are distributed equally to all the classes, whereas the rare N-Grams belong in most of the documents of a specific class. The sparse N-Grams occurred less frequently in the documents of a class, and their presence or absence is not important to decide the class label of the documents. In this paper, we have focused on the selection of most discriminating N-Grams by reducing the effects of symmetrical distribution. The symmetrical distribution of the N-Grams in more than one class requires computation of the symmetrical information associated with all the classes for the N-Gram. In this paper, we focused on the selection of most discriminating N-Grams by reducing the effects of symmetrical distribution. In this context, a new text feature selection method named as the symmetrical strength of N-Grams (SSNG) is proposed using a two pass filtering based feature selection (TPF) approach.

The two levels of filtering gives better results in our day to day life problems motivated us to develop an approach which filters the text document features in two levels. Initially, the SSNG choose various informative N-Grams as a set *NG* from the entire extracted N-Grams of the corpus (*D*), such that $NG \in D$. In the second pass filtering, benchmarked $\chi ^2$ method (Manning et al. 2008) is being used to select few most informative N-Grams (say $NG[k]\in NG$) from set *NG*. The SSNG computes the symmetrical strength of the N-Grams based on four criteria- symmetrical uncertainty, membership, strength, and the nature of the N-Gram. To evaluate the performance of the SSNG using TPF approach, we have conducted a substantial number of experiments on movie review (Pang and Lee 2004), ACL IMDB (Maas et al. 2011), Reuters13 (Forman 2003), 20Newsgroup (Joachims 1996), Ohsumed5, Ohsumed10, Ohsumed23 (Joachims 1998) and Pubmed9 data sets using two standard classifiers Multinomial Naive Bayes (MNB) and linear Support Vector Machine (LSVM). In most of the data sets the performance and success rate of the proposed SSNG method using TPF approach is superior to the state-of-the-art methods viz. MI, IG, OR, DFS, and $\chi ^2$.

The remaining part of the paper is organized as follows: The preliminary concepts are discussed in “Preliminary concept” section. The related works are described in “Related works” section. “Proposed work” section describes the proposed work. “Results and discussions” section illustrates results and discussion. The paper is concluded in the “Conclusion” section.

## Preliminary concept

The preliminary concept is discussed in this section to explain the contribution part of this study. The preliminary notations are described in Table 1.

**Table 1:** The preliminary notations

Notations	Formula	Meaning
*a*	$=Count(NG_{i}|C_{j})$	The count of the N-Gram $NG_{i}$ when it occurs in the documents of class $C_{j}$
*b*	$=Count(\bar{NG}_{i}|C_{j})$	The count of other the N-Grams $\bar{NG}_{i}$ occurred in the documents of class $C_{j}$
*c*	$=Count(NG_{i}|\bar{C}_{j})$	The count of the N-Gram $NG_{i}$ occurred in the documents of other classes $\bar{C}_{j}$
*d*	$=Count(\bar{t}_{i}|\bar{C}_{j})$	The count of other the N-Grams $\bar{t}_{i}$ occurred in the documents of other classes $\bar{C}_{j}$
*N*	$= (a+b+c+d)$	The total number of N-Grams occurred the documents of all the classes
$p(NG_{i})$	$=(a+c)/N$	The probability of the N-Gram $NG_{i}$
$p(\bar{NG}_{i})$	$=(b+d)/N$	The probability of other the N-Grams $\bar{NG}_{i}$
$p(C_{j})$	$=(a+b)/N$	The probability of the class $C_{j}$
$p(\bar{C}_{j})$	$=(c+d)/N$	The probability of other classes $\bar{C}_{j}$
$p(NG_{i},C_{j})$	$=a/N$	The probability of the N-Gram $NG_{i}$ for being in the class $C_{j}$
$p(\bar{NG}_{i},C_{j})$	$=b/N$	The probability of other N-Grams $\bar{NG}_{i}$ for being in the class $C_{j}$
$p(NG_{i},\bar{C}_{j})$	$=c/N$	The probability of the N-Gram $NG_{i}$ for being in other classes $\bar{C}_{j}$
$p(\bar{NG}_{i},\bar{C}_{j})$	$=d/N$	The probability of other N-Grams $\bar{t}_{i}$ for being in other classes $\bar{C}_{j}$
$p(NG_{i}|C_{j})$	$=a/(a+b)$	The probability of the N-Gram $NG_{i}$ when it co-occurs with class $C_{j}$
$p(\bar{NG}_{i}|C_{j})$	$=b/(a+b)$	The probability of other N-Grams $\bar{t}_{i}$ when they co-occur with the class $C_{j}$
$p(NG_{i}|\bar{C}_{j})$	$=c/(c+d)$	The probability of the N-Gram $NG_{i}$ when it co-occur with other classes $\bar{C}_{j}$
$p(\bar{NG}_{i} | \bar{C}_{j})$	$=d/(c+d)$	The probability of other N-Grams $\bar{t}_{i}$ when they co-occur with other classes $\bar{C}_{j}$
$p(C_{j}|NG_{i})$	$=a/(a+c)$	The probability of class $C_{j}$ when the N-Gram $NG_{i}$ co-occurs with the class $C_{j}$
$p(C_{j}|\bar{NG}_{i})$	$=b/(b+d)$	The probability of the class $C_{j}$ when other N-Grams $\bar{NG}_{i}$ co-occur with class $C_{j}$
$p(\bar{C}_{j}|NG_{i})$	$=c/(a+c)$	The probability of other classes $\bar{C}_{j}$ when the N-Gram $NG_{i}$ co-occur with other classes $\bar{C}_{j}$
$p(\bar{C}_{j}|\bar{NG}_{i})$	$=d/(b+d)$	The probability of other classes $\bar{C}_{j}$ when other N-Grams $\bar{NG}_{i}$ co-occur with other classes $\bar{C}_{j}$

### Term representation

**Fig. 2:**
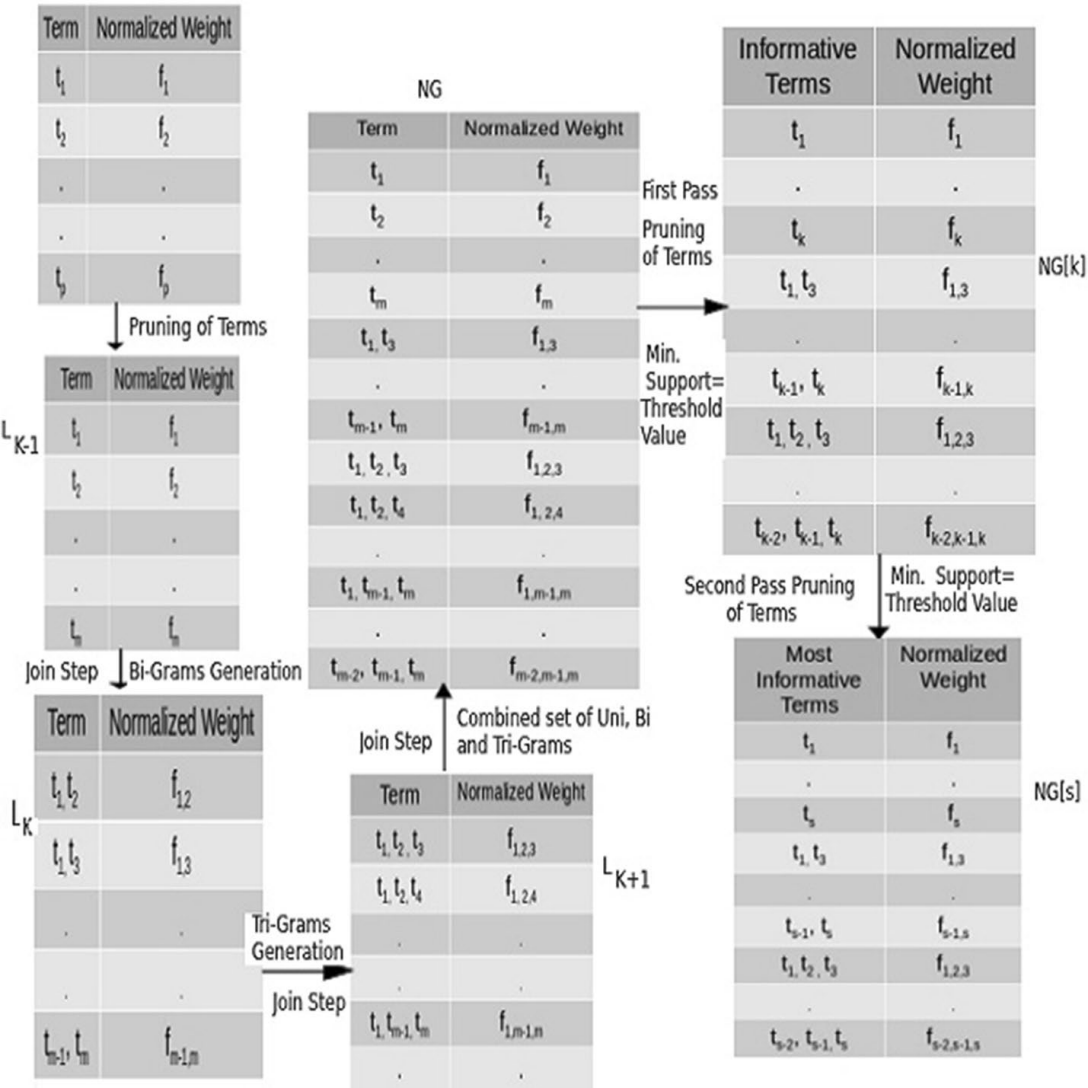
The most informative frequent N-Grams mining

In this paper, we adopted NGL model to represent the terms as a single set of N-Grams, *NG*, by combining the set of Uni, Bi, and Tri-Grams (see Fig. 2). The set *NG* and its subsets *NG*[*k*] and *NG*[*s*] have been generated by the Apriori algorithm.

To find the frequent terms occurred together in the sentences of various class documents a two-step process, **join** and **prune**, have been employed.

*1. The join step:* This step generates a new list of terms $L_{k}$ which is the combination of terms of set $L_{k-1}$ by joining it with itself, i.e., $L_{k-1} \bowtie L_{k-1}$. E.g., $L_{k}$ is a set of Bi-Grams, represented as $L_{k}=\{t_{1} t_{2},..,t_{m-1}t_{m}\}$. It is generated by making the ordered pair of each term of Uni-Grams set $L_{k-1}=\{t_{1},t_{2},..,t_{m}\}$, i.e., $(t_{m-1},t_{m})$ where $t_{m-1},t_{m}\in L_{k-1}$. Similarly, the set of Tri-Grams $L_{k+1}$ has been generated. It is the ordered triplet of terms of $L_{k-1}$, i.e., $L_{k+1}=\{t_{1}t_{2}t_{3},..,t_{m-2}t_{m-1}t_{m}\}$. Finally, the set *NG* is generated by taking the union of Uni, Bi, and Tri-Grams set, i.e., $L_{k+1}\bigcup L_{k}\bigcup L_{k-1}$.

*2. The prune step:* This step eliminates some of the unimportant N-Grams from the set *NG* by using a threshold value. Here, the elimination is based on the weight of the N-Gram. The proposed $\hbox {SSNG} + \chi ^2$ method is used to select the most informative N-Grams set *NG*[*k*], such that $NG[k]\subset NG$.

## Related works

In literature many researchers have significantly contributed in this direction and compared their core contributions with state-of-the-art methods viz. MI, IG, OR, DFS, $\chi ^2$ and TF-IDF. We described the brief description about these methods in this section.

The Mutual information (MI) concept (Manning et al. 2008; Joachims 1998) has been carried out from the information theory to measure the dependencies between random variables and used to measure the information contained by an N-Gram $NG_{i} \in NG$ (see Eq. 1). It is strongly influenced by the marginal probabilities of the N-Grams. It assigns higher weight to the rare N-Grams than common and sparse N-Grams. Therefore the N-Grams weights are not comparable for the N-Grams with widely differing frequencies (Wang et al. 2014; Yang and Pedersen 1997).

1 $$\begin{aligned} MI(NG_{i},C_{j}) = \sum _{NG=1,C=1}^{NG=size(NG),C=r} p(NG_{i}, C_{j}) \times \left[ \log { \frac{p(NG_{i}, C_{j})}{p(NG_{i})\times p(C_{j})}}\right] \end{aligned}$$

The Information Gain (IG) is a measure of reduction in entropy for the N-Grams when they are separated into different classes. The IG assigns higher weight to common N-Grams distributed in many categories than rare N-Grams. The IG is also known as average MI. The computation of IG includes the estimation of the conditional probabilities of a category given an N-Gram and its entropy (see Eq. 2). It is the difference between the original information requirement (i.e. based on the proportion of classes) and the new requirement (i.e., obtained after partitioning of N-Gram $NG_{i}$) (Wang et al. 2014; Uysal and Gunal 2012; Forman 2003; Yang and Pedersen 1997; Lewis and Ringuette 1994).

2 $$\begin{aligned} IG(NG_{i},C_{j})& = -\sum \limits _{C=1}^{C=r} p(C_{j})\log {p(C_{j})} \nonumber \\& \quad + p(NG_{i}) \times \left[ \sum \limits _{NG=1,C=1}^{NG,C=r} (p(C_{j}|NG_{i}) \times \log {(p(C_{j}|NG_{i})}\right] \nonumber \\&\quad + p(\bar{NG}_{i}) \times \left[ \sum \limits _{NG=1,C=1}^{NG,C=r} (p(C_{j}|\bar{NG}_{i}) \times \log {(p(C_{j}|\bar{NG}_{i})} \right] \end{aligned}$$

The Odds ratio (OR) was originally proposed by Rijsbergen (1979) to select the N-Grams for relevance feedback. The OR method is a one sided local feature selection method (Uysal 2016). It is the ratio of the odds of an N-Gram $NG_{i}$ occurring in a class $C_{j}$ to its odds in other classes $\bar{C_{j}}$ (see Eq. (3)). It is based on the assumption that, the distribution of the features on the relevant documents varies from non-relevant documents. Mladenic and Grobelnik (1999) used OR method and achieved highest F1-measure using MNB classifier.

3 $$\begin{aligned} OR(t_{i},C_{j})= \sum _{NG=1,C=1}^{NG,C=r} \log {_{2} \left[ \frac{(p(NG_{i} | C_{j}) \times (1- p(NG_{i} | \bar{C}_{j})}{(p(NG_{i} | \bar{C}_{j}) \times (1- p(NG_{i} |C_{j})}\right] } \end{aligned}$$

Uysal and Gunal (2012) defined the Discriminating Feature Selector (DFS) method to compute the weight of an N-Gram $NG_{i}$ for a class $C_{j}$ (see Eq. 4). The DFS is an improvement of the MI by reducing the effect of marginal probabilities of the N-Grams by normalizing the weight. The DFS defines four categories of N-Grams. It assigns weight of the N-Grams in the range of [0.5,1].

4 $$\begin{aligned} DFS(NG_{i},C_{j}) = \sum _{NG=1,C=1}^{NG,C=r} \frac{p(C_{j} |NG_{i})}{p(\bar{NG_{i}}|C_{j})+p(NG_{i}|\bar{C_{j}})+1} \end{aligned}$$

Mathematically, Chi-square (Manning et al. 2008) testing is used to determine the independence of the term $NG_{i}$ and class $C_{j}$ during the feature selection (see Eq. 5). The $\chi ^2$ method assigns higher weight to common N-Grams than rare N-Grams. It is better than MI because it assigns normalized weight to the terms. Therefore $\chi ^2$ weighted terms are comparable in the same category. However, this normalization breaks down for low frequency terms & it is not reliable for low frequency terms (Wang et al. 2014; Yang and Pedersen 1997).

5 $$\begin{aligned} \chi ^2(NG_{i},C_{j})= \sum \limits _{NG=1,C=1}^{NG,C=r} \frac{N\times (a \times d-b \times c)^{2}}{(a+c)\times (a+b)\times (c+d)\times (b+d)} \end{aligned}$$

Guo et al. (2009) achieved 83.0 % f1 by using self-switching classifier, while 67.7 and 74.7 % f1 using SVM and MNB in 20Newsgroup datasets (10 number of categories were taken). In Ohsumed15 dataset this self-switching classifier gains 73.9 % f1, while 70.2 and 70.9 % using SVM and MNB.

Rehman et al. (2015) achieved peak macro f1 by 21.07 % (for 1500 features) using LSVM in Ohsumed23 dataset. In 20Newsgroup dataset his proposed method gain 74.38 % macro f1 while 75.54 % micro f1 using LSVM, similarly 72.99 % macro and 73.10 micro f1 using MNB.

Uysal (2016) proposed an improved global feature selection scheme for text classification. It is an ensemble method combining the power of two filter-based methods. The new method combines a global and a one-sided local feature selection method. By incorporating these methods, the feature set represents classes almost equally. This method outperforms the individual performances of feature selection methods.

Sharma and Dey (2012) reviewed extensively on sentiment classification problem and described year wise research findings of authors, models with accuracy on review datasets. The maximum 95 % accuracy had been achieved by the authors in the movie review dataset.

## Proposed work

### The SSNG method

The symmetrical strength of the N-Gram ($NG_{SSNG}$) is based on four criteria- symmetrical uncertainty ($NG_{SU}$), membership ($NG_{Mem}$), strength ($NG_{Strength}$), and the nature of the terms ($NG_{RCST}$).

6 $$\begin{aligned} NG_{SSNG} =\sum _{NG=1,C=1}^{NG,C=r}\left( NG_{SU} + NG_{Mem} + NG_{Strength} \right) ^3 \times (NG_{RCST})^4 \end{aligned}$$

*The Symmetrical Uncertainty of the N-Grams *($NG_{SU}$*)* The ratio of the information gain of the $i$th N-Gram $NG_{i}$ for the class $C_{j}$ with the sum of probabilities of $NG_{i}$ and class $C_{j}$ reduces the symmetrical uncertainty of the N-Gram. If the information gain of the $i$th N-Gram $NG_{i}$ is very high due to high frequency of the common or sparse N-Gram then by dividing this information gain value with the sum of probabilities of N-Gram and the class will be reduced to a smaller value (see Eq. (7)).

7 $$\begin{aligned} NG_{SU}(NG_{i},C_{j}) =2 \times \frac{ IG(NG_{i},C_{j})}{p(NG_{i})+p(C_{j})} \end{aligned}$$

*The Membership of the N-Gram in a class (*$NG_{mem}$*)* The belongings of the N-Gram to the specific class is referred as membership of the N-Gram. A probabilistic ratio of success or failure is computed to evaluate whether the N-Gram belongs to a specific class or not (see Eq. (8)).

According to the criteria used by Uysal and Gunal (2012), the N-Gram present in only one class is more important than others. The minimum N-Gram frequency of such N-Grams in a class is zero. Dividing the numerator of the Eq. (8) by such type of N-Grams will produce an undefined number. Therefore, a very small number $\epsilon$ which is closer to zero, but not zero ( $0< \epsilon <= 0.5$) has been added in the numerator and denominator of the Eq. (8) to avoid the division by zero error.

The Eq. (8) for computing the membership of $NG_{i}$ in a class $C_{j}$ is similar to the OR (see Eq. (3)). In case of two class problems, the OR assigns equal positive and negative weights to the N-Gram $NG_{i}$ for the class $C_{j}$ and other classes $\bar{C}_{j}$. It is due to its one sided weight computation nature. In case of multi-class problems, although the weight assignment of the OR is not equal for all the classes, but due to its one sided nature the positive and negative weights of the N-Gram for different classes have less discriminating power. The extra $\epsilon$ has been added in the OR method before taking the logarithm to boost the score of such type of N-Grams which are present only in one class.

8 $$\begin{aligned} NG_{Mem}(NG_{i},C_{j})= \log {_{2}\left[ \epsilon +\frac{\epsilon +(a \times d)}{\epsilon +(b \times c)}\right] } \end{aligned}$$

*The Strength of an N-Gram (*$NG_{Strength}$*)* It is an improvement of the standard mutual information (Forman 2003) method (see Eq. 1), where each logarithmic quantity is multiplied by $P(NG_{i},C_{j})$ (see Table 1). The computation of $NG_{Strength}$ of the term $NG_{i}$, each logarithmic quantity is multiplied with the total occurrence of term $NG_{i}$ in the documents of class $C_{j}$ and other classes $\bar{C}_{j}$ (see Eq. 9).

9 $$\begin{aligned} NG_{Strength}(C_{j}|NG_{i}) = Count(NG_{i},C_{j}) \times \left[ \log {\frac{p(NG_{i},C_{j})}{p(NG_{i})\times p(C_{j})}}\right] \end{aligned}$$

*The nature of the N-Gram (*$NG_{RCST}$*)* The absolute difference between the probabilities of the class $C_{j}$ and other classes $\bar{C}_{j}$ when the $i$th N-Gram $NG_{i}$ is present, computes the nature of the rare, common, or sparse N-Grams (see Eq. (10)).

10 $$\begin{aligned} NG_{RCST}(NG_{i},C_{j})= |p(C_{j}|NG_{i})-p(\bar{C}_{j}|NG_{i})| \end{aligned}$$

If $NG_{RCST}$ value of the $i\mathrm{th}$ N-Gram $NG_{i}$ is zero or very small then the $NG_{i}$ occurred either equally or less frequently in the documents of all the classes. It means the nature of the N-Gram is either common or sparse. If $NG_{RCST}$ value is high, then the $NG_{i}$ occurred more in one category compared to other categories.

The common and sparse N-Grams are with a low membership value to the specific class, less responsible in exact discrimination of the class of documents. Whereas, the rare N-Grams are with a high membership value to the specific class, more responsible. We have observed from an extensive number of experiments that, the cube of $(NG_{SU} + NG_{Mem} + NG_{Strength})$ instead of square or fourth power, gives maximum accuracy. The fourth power of $NG_{RCST}$, reduces the weight of common and spare N-Grams such as near to the value of zero, whereas, it increases the weight of the rare N-Grams very high in comparison to the benchmarked methods. Therefore the most informative rare N-Grams are selected and the uninformative common and sparse N-Grams are eliminated, if the threshold value represents the top most informative N-Grams. Further, the concept has been explained in the “Illustration of the SSNG using example datasets” section by using two example datasets shown in Tables 2 and 5.

**Table 2:** Example dataset words in category *C*1 and *C*2

Category	N-Gram	Documents					
		D1	D2	D3	D4	D5	D6
C1	“penalty shootout”	0	0	0	0	0	0
	“penalty corner”	1	1	0	1	2	0
	“beautifully”	0	1	1	2	0	1
	“play”	1	1	1	2	2	1

		D7	D8	D9	D10	D11	D12
C2	“penalty shootout”	1	2	0	0	0	1
	“penalty corner”	0	0	0	0	1	0
	“beautifully”	1	0	1	2	0	0
	“play”	0	0	2	0	1	1

### Illustration of the SSNG using example datasets

To further illustrate this concept, consider an example dataset shown in Table 2. We illustrate the process of weight calculation using SSNG method for four N-Grams {“penalty corner”, “penalty shootout”, “beautifully”, “play”} of this example dataset. We assumed, the N-Grams are contained by twelve documents of a balanced dataset with two classes, where each class having six documents (see Table 3). Table 4 shows the confusion matrix of N-Gram “penalty shootout” for its presence or absence to a class $C_{1}$ or in $C_{2}$. The computation of weight for N-Gram “penalty shootout” is as follows-

1. The symmetrical uncertainty has been computed using Eq. (7) as:
  $$\begin{aligned} NG_{SU}(``penalty\ shootout'',C_{1})& = 0.724,\\ NG_{SU}(``penalty\ shootout'',C_{2}) & = 0.724 \end{aligned}$$
2. The Strength of the N-Gram for class *C*1 and other class *C*2 is computed using Eq. (9).
  $$\begin{aligned} NG_{Strength}(C1|``penalty\ shootout'')& = 0 \\ NG_{Strength}(C_{2}|``penalty\ shootout'')& = 5.5911 \end{aligned}$$
3. The membership of the N-Gram for class *C*1 and *C*2 using Eq. (8).
  $$\begin{aligned}&NG_{Mem}(``penalty\ shootout'',C_{1}) \\&\quad = \log _{2}{\left( 0.5+\frac{0.5+0 \times 9}{0.5 + 4 \times 18}\right) } \\&\quad =-0.9802 \\&NG_{Mem}(``penalty\ shootout'',C_{2}) \\&\quad = \left[ \log _{2}{ \left( 0.5+\frac{0.5+4 \times 18}{0.5 + 0 \times 9}\right) }\right] _{j=C2} \\&\quad =7.1849 \end{aligned}$$
4. The nature of the N-Gram for class *C*1 and *C*2 using Eq. (10).
  $$\begin{aligned} NG_{RCST}(``penalty\ shootout'',C_{1})& = 0.8889 \\ NG_{RCST}(``penalty\ shootout'',C_{2})& = 0.8889 \end{aligned}$$
5. Further, we compute the SSNG score of the N-Gram for class *C*1 and *C*2 using Eq. (6).
  $$\begin{aligned}&NG_{SSNG}(``penalty\ shootout'',C_{1}) \\&\quad = \left( (0.724 + 0 - 0.9802)^3\times (0.8889)^4\right) \\&\quad = \left( (-0.2562)^3\times (0.8889)^4\right) =(-0.0168 \times 0.6243) \\&\quad =-0.0105 \\&NG_{SSNG}(``penalty\ shootout'',C_{2}) \\&\quad = \left( (0.724 + 4.983 + 7.1849)^3\times (0.8889)^4\right) \\&\quad = \left( (12.8919)^3\times 0.6243 \right) \\&\quad =(2142.6417 \times 0.6243)=1337.6407 \end{aligned}$$
6. Finally, we compute the total contribution of N-Gram in the classification of text documents as:
  $$\begin{aligned}&NG_{SSNG}(``penalty\ shootout'') \\&\quad = NG_{SSNG}(``penalty\ shootout'',C_{1})\\&\qquad + NG_{SSNG}(``penalty\ shootout'',C_{2}) \\&\quad = -0.0105 + 1337.6407 = 1337.6302 \end{aligned}$$

In this study, we have two main objectives: First, to assign highest weight to the rare N-Grams like “penalty shootout” which appeared only in the class “C2” and “penalty corner” which appeared in the 4 documents of the class “C1” and only once in the document of class “C2”. The second objective is, assigning very less weight to the common N-Grams like “beautifully” and “play”. Here “beautifully” is more informative than “play”, because the document frequency of the “beautifully” is 6 in the class “C1” whereas “play” have 4 only. The document frequencies of both N-Grams in the class “C2” are equal to 3. The SSNG method assigns very less weight to the sparse N-Grams. The SSNG method assigns highest weight to N-Gram “penalty shootout” = 1337.6302. The other feature selection methods also give more score to this N-Gram, but the computed weight by the SSNG is very high. The similar calculation of the SSNG weight for other N-Grams gives scores for other N-Grams “penalty corner”= 20.7158, “play”= 0.0.0004, and “beautifully” = 0.3527 ( see Table 4). This example dataset is not normalized because it is very small and contains only four N-Grams in the 12 documents of the two classes. In case of real datasets the terms weigh is normalized using TF-IDF weight before further processing.

**Table 3:** Confusion matrix for N-Gram by class frequency

N-Grams	Class $C_{1}$	Class $C_{2}$
$NG_{penalty\ shootout}=1$	$a=0$	$b=4$
$NG_{penalty\ shootout}=0$	$c=18$	$d=9$

**Table 4:** N-gram scores versus feature selection methods in Example Dataset

Metrics	Penalty shootout	Penalty corner	Beautifully	Play
MI	0.1607	0.1262	0.0939	0.0210
IG	0.1985	0.1129	0.1842	0.1413
OR	0	4.2857	1.7561	0.8491
DFS	0.6391	0.5752	0.5334	0.5257
χ^2^	6.3589	2.3881	0.9297	0.1131
SSNG	*1337.6302*	*20*.*7158*	*0.3527*	*0.0004*

**Table 5:** The representation ability of the N-Grams for the class

N-Grams	Class C1	Class C2	Difference (D)	$D^2$	$D^3$	$D^4$	Nature of the N-Gram
$t_{i}$	2.3	2.25	0.05	0.0025	0.000125	0.00000625	Common
$t_{j}$	2.5	0.1	2.4	5.76	13.824	33.1776	Rare
$t_{k}$	2.5	0	2.5	6.25	15.625	39.0625	Very rare
$t_{l}$	0.05	0.01	0.04	0.0016	0.000064	0.00000256	Sparse

The main aim of taking the cube of $( NG_{SU} + NG_{Mem} + NG_{Strength} )$ is quite clear from the computational process of the SSNG. The power of this quantity can be an odd number (i.e., 1, 3, 5,…) because if we take an even number, it will make the weight of the N-Gram positive for some classes which is currently being assigned a negative value. The discriminating power of these N-Grams is less for that class. The positive and negative combination of the weights for an N-Gram finds more appropriate discriminating power of the N-Gram, instead of positive combinations. It is because, e.g. a rare N-Gram which is present in a specific class $C_{j}$ and absent in other classes, then its positive value for other classes $\bar{C}_{j}$ create ambiguity and will deficit its discriminating power. Further, if we choose the power as one, it will not fulfill our objectives and the weights are computed as similar to the state-of-the-art methods. Further, if we select power more than three, the weights are very high for rare N-Grams as it is already high if we choose it three.

Similarly, $(NG_{RCST})^4$ finds the representation ability of the N-Gram for a class compared to other classes. It will assign the highest weight to the rare, less weight to the common, and very less weight to the sparse N-Grams. Suppose, we have four N-Grams $NG_{i}, NG_{j}, NG_{k}$ and $NG_{l}$ of a example dataset shown in Table 5. The nature of the $NG_{i}$ is common and the other N-Grams $NG_{j}$, $NG_{k}$ and $NG_{l}$ have rare, very rare, and sparse natures respectively. The representation ability of the $NG_{i}$ for a class $C_{1}$ is 2.3 and for other classes $\bar{C}_{1}$ is 2.25 (see Table 5). The absolute difference between the representation ability of the $NG_{i}$ for a class $C_{j}$ and other classes $\bar{C}_{j}$ have been computed to identify the discriminating nature of the $NG_{i}$ in ATDC. In this particular case, we get this absolute difference as $|2.3-2.25|=.05$. The fourth power of $(0.05)^4$ is very small in comparison to $(0.05)^1,(0.05)^2$,and $(0.05)^3$. The fourth power has reduced the weight of common and sparse N-Grams near to zero, whereas increased the weight of the rare N-Grams four times (see Table 5). Therefore, to fulfill our objectives of assigning very less weight to common and sparse N-Grams whereas highest weight to rare N-Grams, we have taken this value as four in $(NG_{RCST})^4$.

We observed that the weight assignment process of the MI, IG, DFS, and $\chi ^2$ are as described in the literature. The MI gives highest weight to rare N-Grams like “penalty shootout” and “penalty corner”, but very less weight (near to zero) to common N-Grams “beautifully” and “play”, which is the cause of its low performance. Similarly, the IG assigns highest weight to “penalty corner” instead of “penalty shootout” and give more weight to “play” than “beautifully”. It is due to its biased nature towards the terms distributed in many categories. Although, its performance is quite better than MI, but performs slightly lower than SSNG & $\chi ^2$.

The DFS assigns highest weight to the rarest N-Grams and minimum weight to the common N-Grams in the range from 0.5 to 1. This method is best suited for the document frequency based weight computations, but does not perform well in case of term frequency based weight computations. The weight assignment process of the $\chi ^2$ based on the term frequency is similar to the SSNG (see Table 4). This is the main reason to select the $\chi ^2$ method, for filtering the SSNG weighted terms, at the second stage.

### The TPF approach

In order to measure the importance of the N-Gram, the SSNG method using the TPF approach is applied. The TPF approach is explained in the Algorithm 1. The TPF Algorithm 1 works as follows:

1. The corpus *D* is divided into two subsets $D_{train}$ and $D_{test}$ in line 1.
2. Subsequently, the function *SECONDPASS(*$D_{train},SSNG,\chi ^2,th1,th2$*)* is called in line 2. This function returns a set *NG*[*s*] of most informative N-Grams (line 31–41).
3. The function *FIRSTPASS(*$D_{train},m1,th1$*)* is called inside *SECONDPASS(*$D_{train},SSNG,\chi ^2,th1,th2$*)* in line 32. It returns the *k* informative N-Grams $NG[k]\subset NG$ (line 20–30). The following functions are called inside *FIRSTPASS(*$D_{train},m1,th1$*)*:
  - *PREPROCESSING(D)* The function in line 21 takes document *D* as an argument and returns the set of tokens *T* after removing stop words, punctuation marks, and white spaces (line 14–19).
  - *COMPUTENGRAM(T)* The function (line 3-8) returns set of N-Grams *NG* in line 22. The Uni-Grams, Bi-Grams , and Tri-Grams are generated in line 4, 5, and 6 respectively. Finally, the set of N-Grams (*NG*) which is the union of Uni-Grams, Bi-Grams, and Tri-Grams have been generated in line 7.
  - The occurrence frequency $NGf_{ij}$ of each N-Gram $NG_{i}$ for each class $C_{j}$ is computed in step 26.
  - *NGSCORE(*$NG_{i},NGf_{ij},f$*)*- It returns a unique weight for $i\mathrm{th}$ N-Gram $NG_{ij}$ of class $C_{j}$ in line 27 using the feature selection methods *f* (MI, IG, OR, DFS, $\chi ^2$, and SSNG) (line 9–13). The total N-Gram frequency $NGf_{ij}$ is the summation of N-Gram frequencies in the documents of the class $C_{j}$.
  - *Sort(*$NG_{i},NGS_{i}$*)* It returns N-Grams after sorting in descending order based on their weights ($NGS_{i}$) in line 28.
  - *Select(FS*[*m*], *threshold)* It returns a set of informative N-Grams from *FS*[*m*] based on a *threshold* value. A numeric threshold value is selected as *th*1 and top *k* N-Grams (*NG*[*k*]) are extracted based on their numeric score (line 29).
4. The TF-IDF weight of all *k* N-Grams (*NG*[*k*]) are computed in line 36.
5. The TF-IDF weighted total N-Gram frequency $NGf_{ij}$ is the summation of N-Gram frequencies ($Count(NG_{i}|C_{j})$) in the documents of the class $C_{j}$ (line 37).
6. The *k* TF-IDF weighted N-Grams are passed into $\chi ^2$ method in line 38 to compute a new numeric score of each N-Gram.
7. The N-Grams are arranged in descending order in line 39 based on new numeric score *newNGS*[*NG*[*k*]] of N-Gram *NG*[*k*].
8. Either all *k* N-Grams or less than *k* (*s*) N-Grams are stored in a set *BFS*[*s*] as most informative N-Grams in line 40.

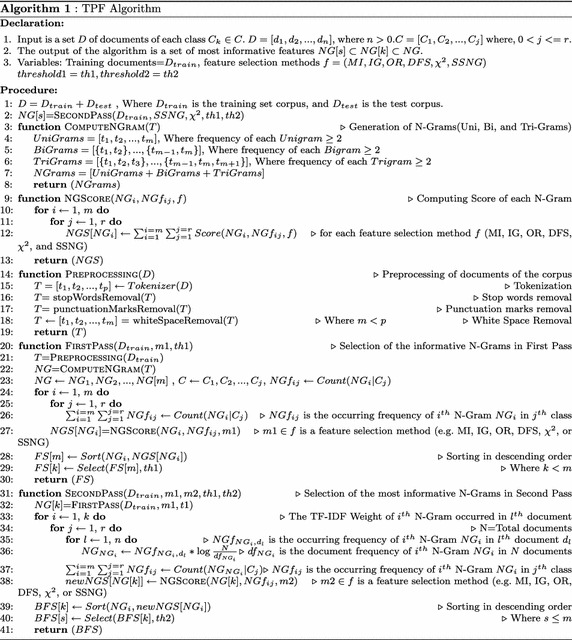


*Time Complexity Analysis of the Algorithm 1* The time complexity of the Algorithm 1 is computed as follows:

1. Let *n* is the total number of documents, *r* is total number of classes, *p* is the total number of terms, *m* number of terms are obtained after removal of stop words, punctuation marks and white spaces, *M* is the total number of N-Grams, *k* numbers of N-Grams are selected as informative N-Grams based on threshold value at first pass, and *s* numbers of N-Grams are selected in the second pass.
2. The generalized formula for computing the total number of N-Grams is:
  11 $$\begin{aligned} M = m + \sum _{j=1}^{j=m}\left( m-j\right) + \sum _{q=1}^{q=\left( m-2\right) }\frac{\left( q^2+q\right) }{2} \end{aligned}$$
  where, *m* is the size of Uni-Grams, $\sum _{j=1}^{j=m}\left( m-j\right)$ is the size of Bi-Grams, and $\sum _{q=1}^{q=\left( m-2\right) }\frac{\left( q^2+q\right) }{2}$ is the size of Tri-Grams.
3. ${\mathcal {O}}(M \times n \times r)={\mathcal {O}}(M)$ time complexity is required to read the *M* number of N-Grams from *n* documents of *r* classes, because *n* and *r* are very less as compared to *M* (from Declaration part to line 1).
4. The loop (line 24–25) requires ${\mathcal {O}}(M \times r)$ time complexity to compute the weight of $NG_{i}$ for the class $C_{j}$.
5. The loop (line 33–35) requires ${\mathcal {O}}(k \times n \times r)$ time complexity to compute the weight of $k$th N-Gram *NG*[*k*] in *n* documents of *r* classes.
6. ${\mathcal {O}}(k\log {}k)$ time complexity is required to sort the *k* N-Grams based on their weights (line 28 & 39).
7. ${\mathcal {O}}(k)$ time complexity is required to select discriminating *k* N-Grams based on threshold value *th*1 & *th*2 (line 29 & 40).
8. The values of *n*, *r*, *s* and *k* are very less compare to *M*, because the total number of N-Grams *M* are in millions and others are in the hundreds or thousands. Thus, the overall time complexity of the Algorithm 1 is computed as ${\mathcal {O}}(M)$.

## Data set

In this study, we have experimented with ten standard text data sets movie reviews, 20Newsgroup, Reuters13, Ohsumed23 and Ohsumed10. We also worked on Pubmed9 dataset, which consists of nine categories. The detailed summary of the data sets used in the study is given in Table 6.

**Table 6:** Details of the experimental datasets

S. No.	Dataset	Categories name	Total number of classes
1.	Movie review	pos, neg	2
2.	ACL IMDB large movie review	pos, neg	2
3.	20Newsgroup	talk.religion.misc, talk.politics.misc, alt.atheism, talk.politics.guns, talk.politics.mideast, comp.os.ms-windows.misc, comp.sys.mac.hardware, comp.graphics, misc.forsale, comp.sys.ibm.pc.hardware, sci.electronics, comp.windows.x, sci.space, rec.autos, sci.med, sci.crypt, rec.sport.baseball, rec.motorcycles, soc.religion.christian, rec.sport.hockey	20
4.	Reuters13	lei, housing, bop, wpi, retail, ipi, jobs, reserves, cpi, gnp, interest, trade, money-fx	13
5.	Ohsumed5	C01, C02, C03, C04, C05	5
6.	Ohsumed10	C01, C02, C03, C04, C05, C06, C07, C08, C09, C10	10
7.	Ohsumed15	C01, C02, C03, C04, C05, C06, C07, C08, C09, C10, C11, C12, C13, C14, C15	15
8.	Ohsumed23	C01, C02, C03, C04, C05, C06, C07, C08, C09, C10, C11, C12, C13, C14, C15, C16, C17, C18, C19, C20, C21, C22, C23	23
9.	Pubmed9	bird flu, swine flu, proteins, cancer, Bacterial Pneumonia, Fungal Pneumonia, Viral Pneumonia, Idiopathic interstitial pneumonia, Legionnaires	9
10.	BBC	business, entertainment, politics, sport, tech	5
11.	BBC_Sports	athletics, cricket, football, rugby, tennis	5

The movie reviews dataset^5^ was prepared by Pang and Lee (2004) and contains movie reviews collected from the http://www.imbdb.com (Internet Movie Data-base).^6^ This dataset has been used as a benchmark by many researchers, and it is also known as polarity dataset v2.0 or Cornell Movie Review Dataset. There are total of 1000 positive and 1000 negative reviews and this dataset is based on two class problem (Sharma and Dey 2012; Pang and Lee 2004).

The ACL IMDB movie review dataset^7^ is a very large dataset for binary sentiment classification containing substantially more data than previous benchmark datasets. In this data set 25,000 highly polar movie reviews for training, and 25,000 for testing (Maas et al. 2011).

The 20Newsgroups(20ng) dataset contains newsgroup documents from 20 different classes (Joachims 1996). The original owner of this dataset was Mitchell (1997). This dataset is known for its large size and balanced classes. This data set consists of 20,000 messages taken from 20 newsgroups.^8^

The Reuters dataset is the most widely used dataset for text classification. The Reuters13 is a subset of the Reuters dataset as used by Forman (2003). It consists of 13 classes out of 90 from the original Reuters dataset.

The Ohsumed dataset^9^^,^^10^ is the challenging dataset due to its very high sparsity (Joachims 1998). There are 23 classes of documents which are combinations of title and abstracts taken from Pubmed. We partitioned this dataset into four sub data sets Ohsumed5, Ohsumed10, Ohsumed15, and Ohsumed23. These sub datasets contain 5, 10, 15 and 23 classes of articles respectively.

The Pubmed9 dataset used in the experimental study is similar in structure to Ohsumed dataset. It contains documents of nine classes. Each document is a combination of abstracts with their title. All the documents are automatically extracted from the Pubmed website using Entrez software utilities^11^ in R environment.^12^ The nine classes of documents for this data set are viz. bird flu, swine flu, proteins, cancer, Bacterial Pneumonia, Fungal Pneumonia, Viral Pneumonia, Idiopathic interstitial pneumonia, Legionnaires. Each class contains 5000 documents on this data set.

The BBC dataset^13^ consists of 2225 documents from the BBC news website, corresponding to stories in five topical areas from the year 2004–2005. It contains 5 Class Labels viz. business, entertainment, politics, sport, and tech (Greene and Cunningham 2006).

The BBC_Sports dataset (Greene and Cunningham 2006) consists of 737 documents from the BBC Sport website corresponding to sports news articles in five topical areas from the year 2004–2005. Their are 5 Class Labels viz. athletics, cricket, football, rugby, and tennis in this dataset.

### Performance evaluation metrics

The computation of the classifier’s performance is based on the Precision (Eq. (12)), Recall (Eq. (13)), F1-measure (Eq. (15)), and accuracy (Eq. (14)) parameters (Sharma and Dey 2012).

12 $$\begin{aligned} Precision= \frac{TP}{TP+FP} \end{aligned}$$

13 $$\begin{aligned} Recall= \frac{TP}{TP+FN} \end{aligned}$$

14 $$\begin{aligned} accuracy= \frac{TP+TN}{(TP+FP+TN+FN)} \end{aligned}$$

15 $$\begin{aligned} f1\_measure= 2\times \frac{Precision \times Recall}{Precision+Recall} \end{aligned}$$

where TP is true positives, FP is false positives, FN is false negatives, and TN is true negatives.

### Experimental setup

All the experiments have been carried out on a machine with specification as core i7, 8GB RAM, 2.4 GHz Processor in UBUNTU 14.04 64-bit OS. We have used R-3.1.2 to automatically extract articles from the Pubmed website, and Mysql 5.6 to store the information related to articles in the database.

The process of ATDC- Tokenization, preprocessing of the words of the corpus (*T*), feature extraction ($NG\supset T$), feature selection ($NG[k]\subset NG$ and $NG[s]\subset NG[k]$), and statistical analysis are performed in Python 2.7 with nltk, scipy, numpy, ipython notebook, scikitlearn, matplotlib etc. packages.^14^ In order to to prepare the Pubmed9 dataset, we used the Entrez software utility,^15^ to fetch the PubMed articles from the NCBI web page.

We experimented on ten standard datasets along with the Pubmed9 dataset. The Apriori algorithm based the TPF approach has been used to select the most informative N-Grams. Initially, the corpus *D* is divided into two subsets training ($D_{train}$) and test ($D_{test}$), tokenized the sentences of the documents into tokens ($t_{p}$), web links, punctuation marks, stop words, and white spaces have been removed. The set of N-Grams *NG* have been generated. In continuation, we choose *k* informative N-Grams ($NG[k]\subset NG$). In the first pass of the TPF approach, we choose *k* as 500, 1000, 2000, 3000, 5000, 10,000, 15,000, and 20,000. Subsequently, the feature selection methods viz. MI, IG, OR, DFS, $\chi ^2$ and SSNG have been applied to select the *k* informative N-Grams. In the second pass, we applied the $\chi ^2$ method which further filters 500, 1000, 2000, 3000, 5000, 10,000, 15,000, and 20,000 N-Grams, and select the most informative N-Grams ($NG[s]\subset NG[k]$), based on the maximum accuracy gained by the MNB and LSVM classifiers.

## Results and discussions

The experimental results have been compared using maximum accuracy achieved by the classifiers MNB and LSVM, based on the most informative N-Grams ($NG[s]\subset NG[k]\subset NG$) selected using $\hbox {MI}+\chi ^2$, $\hbox {IG}+ \chi ^2$, $\hbox {OR}+ \chi ^2$, $\hbox {DFS}+ \chi ^2$, $\chi ^2+ \chi ^2$, and $\hbox {SSNG} + \chi ^2$. We have performed eight experimental trials for both the classifiers MNB and LSVM. The experimental trials are based on the selection of most informative N-Grams as 500, 1000, 2000, 3000, 5000, 10,000, 15,000, and 20,000 (eight for each classifier). Finally, their are total sixteen experimental trials for each dataset. The success rate of the classifiers in each dataset is based on these experimental trials.

**Fig. 3:**
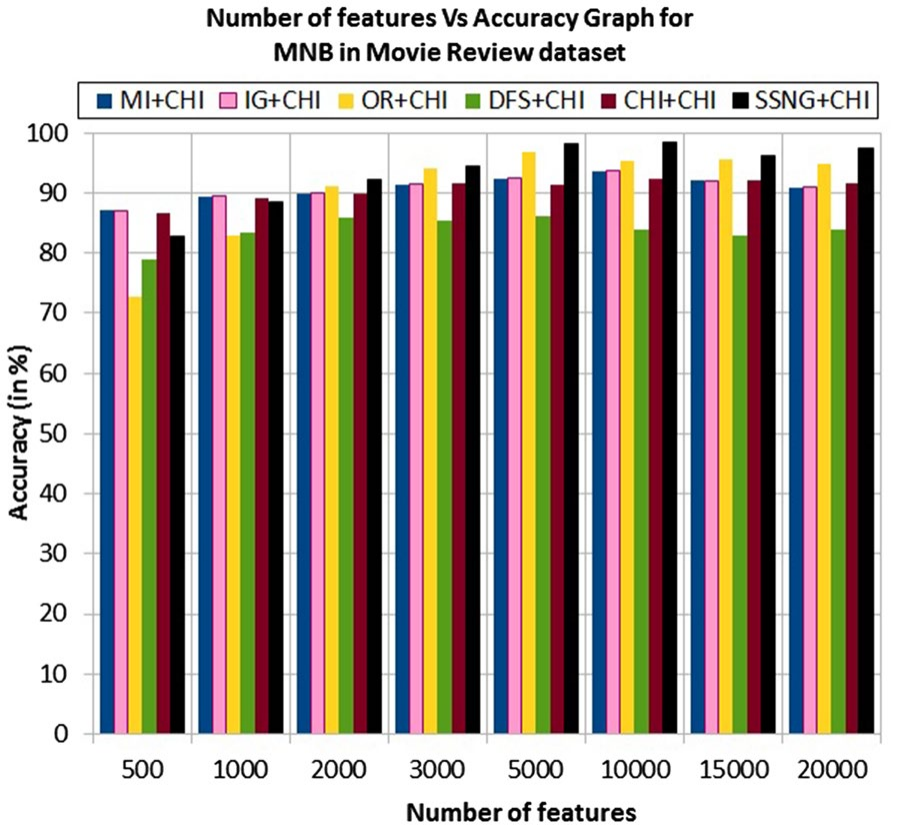
MNB on movie review Dataset

**Fig. 4:**
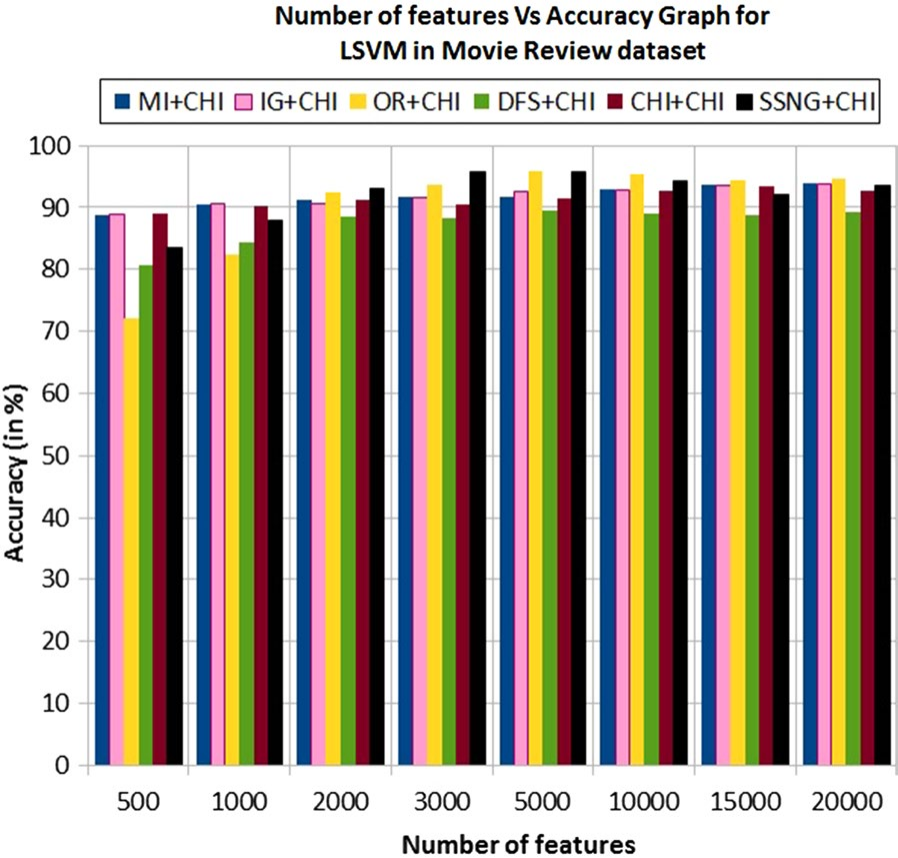
LSVM on movie review Dataset

In the movie review dataset, the accuracy of the MNB classifier depends upon the number of features and achieves the peak value 98.4 % for 10,000 numbers of features (see Table 7) then decreases and remain constant (see Fig. 3). In case of LSVM, the SSNG gains highest 95.8 % accuracy for 3000 and 5000 numbers of features (see Table 7) then decreases and remain constant (see Fig. 4). The success rate of SSNG based on the TPF approach in the movie review dataset is 56.25 % because out of 16 experiments 9 times the $SSNG \, +\chi ^2$ method performed better compared to other methods.

**Table 7:** Performance rank of TPF based methods in six datasets

Classifier	S. No.	Dataset	Maximum accuracy achieved (%)	Number of features	Method
MNB	1.	movie review	98.4	10,000	$\hbox {SSNG} + \chi ^2$
	2.	ACL IMDB	89.81	20,000	$\hbox {SSNG} + \chi ^2$
	3.	Ohsumed5	84.03	1000	$\hbox {SSNG} + \chi ^2$
	4.	Ohsumed10	67.32	2000	$\hbox {SSNG} + \chi ^2$
	5.	Ohsumed15	43.91	2000	$\hbox {SSNG} + \chi ^2$
	6.	Ohsumed23	43.91	2000	$\hbox {SSNG} + \chi ^2$
	7.	Pubmed9	73.84	5000	$\hbox {SSNG} + \chi ^2$
	8.	20Newsgroup	95.6	500	$\chi ^2+ \chi ^2$
	9.	Reuters13	71.59	500	$\chi ^2+ \chi ^2$
	10.	BBC_Sports	98.39	500, 1000, and 2000	$\hbox {SSNG} + \chi ^2$
	11.	BBC	99.28	1000, 5000	$\hbox {IG} + \chi ^2$, $\hbox {SSNG} + \chi ^2$
LSVM	1.	movie review	95.8	3000, and 5000	$\hbox {SSNG} + \chi ^2$, and $\hbox {SSNG} + \chi ^2$, $\hbox {OR} + \chi ^2$
	2.	ACL IMDB	89.94	15,000	$\hbox {SSNG} + \chi ^2$
	3.	Ohsumed5	86.24	3000,10,000	$\hbox {SSNG} + \chi ^2$
	4.	Ohsumed10	70.18	15,000	$\hbox {SSNG} + \chi ^2$
	5.	Ohsumed15	65.75	10,000	$\hbox {SSNG} + \chi ^2$
	6.	Ohsumed23	48	15,000	$\hbox {SSNG} + \chi ^2$
	7.	Pubmed9	74.15	2000	$\hbox {SSNG} + \chi ^2$
	8.	20Newsgroup	95.8	3000, and 5000	$\hbox {SSNG} + \chi ^2$
	9.	Reuters13	78.52	2000	$\hbox {SSNG} + \chi ^2$
	10.	BBC_Sports	100	500, 1000, and 3000	$\chi ^2+ \chi ^2$, $\hbox {IG} + \chi ^2$, and $\hbox {SSNG} + \chi ^2$
	11.	BBC	99.64	10,000	$\hbox {SSNG} + \chi ^2$

**Fig. 5:**
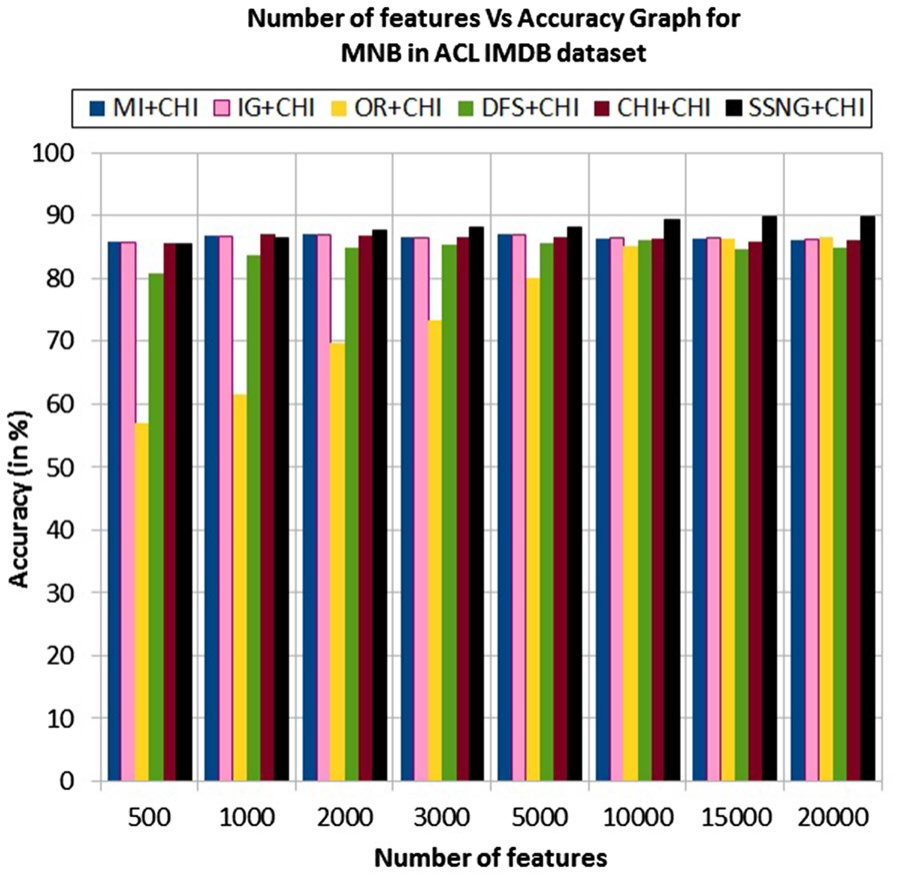
MNB on ACL IMDB large movie review dataset

**Fig. 6:**
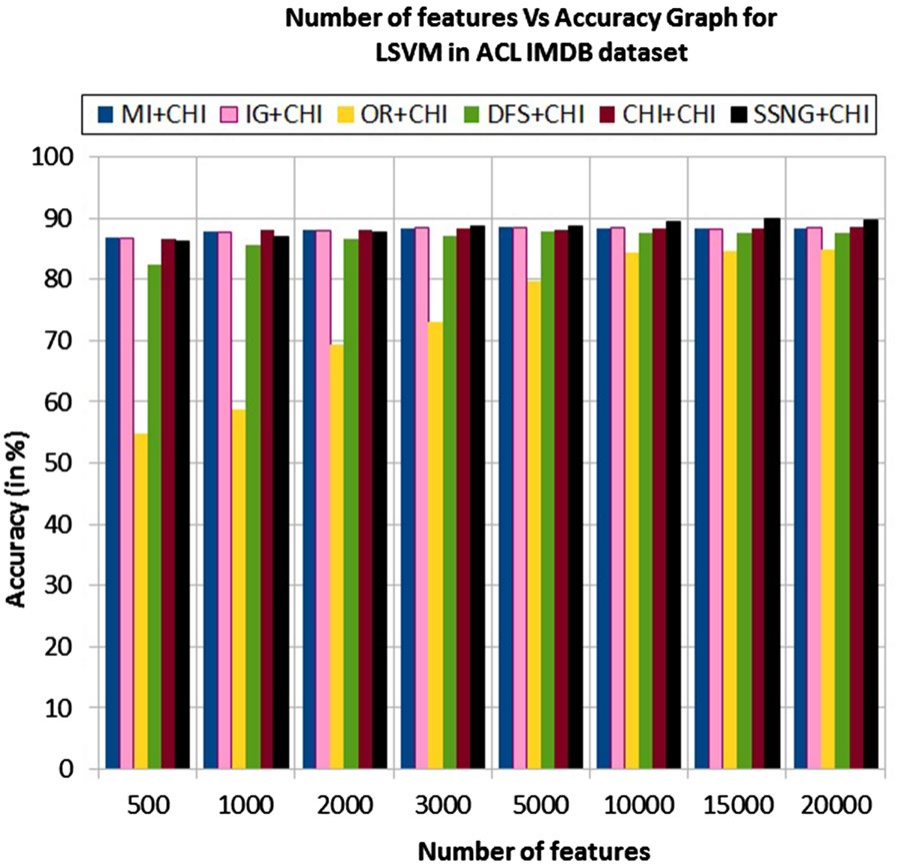
LSVM on ACL IMDB large movie review dataset

In the ACL IMDB dataset, the accuracy of the MNB classifier depends upon the number of features and achieves the peak value 89.81 % for 20,000 numbers of features (see Table 7) then decreases and remain constant (see Fig. 5). In case of LSVM, the SSNG gains highest 89.94 % accuracy for 15,000 numbers of features (see Table 7) then decreases and remain constant (see Fig. 6). The success rate of SSNG in ACL IMDB large movie review dataset is 68.75 % because out of 16 experiments 11 times the $\hbox {SSNG} + \chi ^2$ method performed better compared to other methods.

**Fig. 7:**
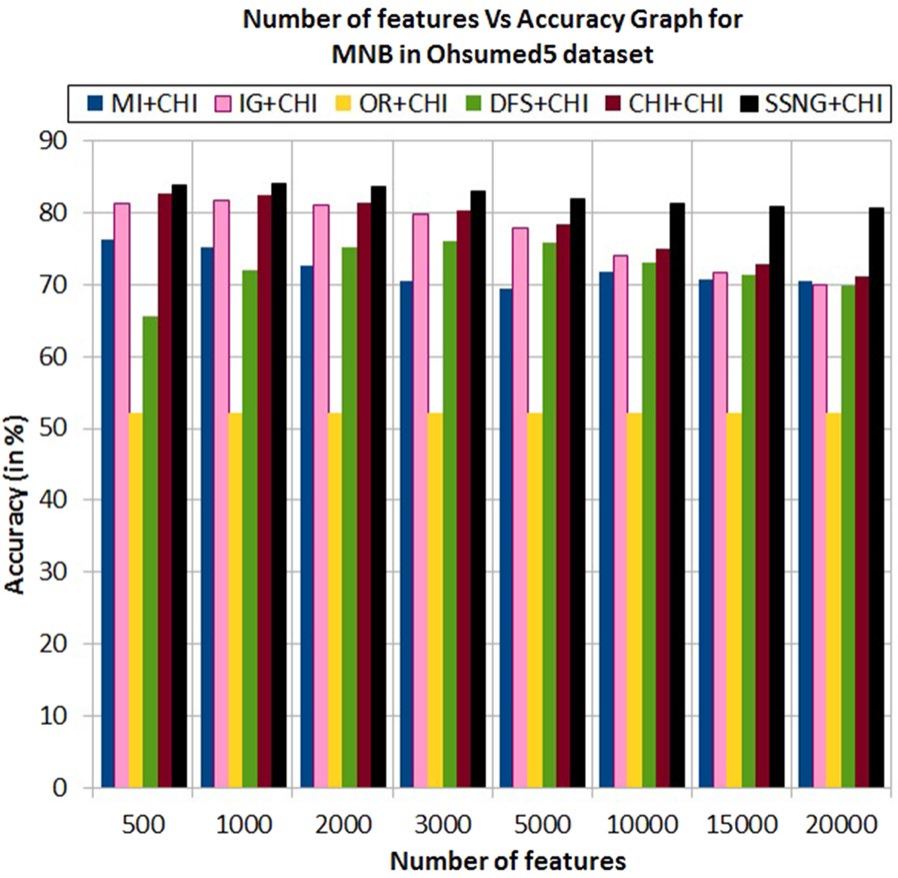
MNB on Ohsumed5 dataset

**Fig. 8:**
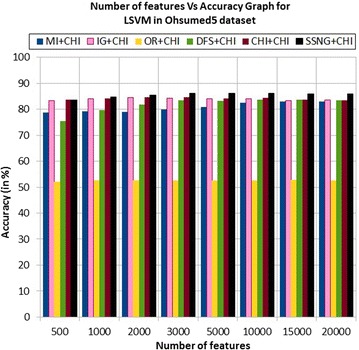
LSVM on Ohsumed5 dataset

In the Ohsumed5 dataset, the accuracy of the MNB classifier depends upon the number of features and achie-ves the peak value 84.03 % for 1000 numbers of features (see Table 7) then decreases and remain (see Fig. 7). In case of LSVM, the SSNG gains highest 86.24 % accuracy for 3000 and 10,000 numbers of features (see Table 7) then decreases and remain constant (see Fig. 8). The success rate of SSNG in Ohsumed5 dataset is 93.75 % because out of 16 experiments 15 times the $\hbox {SSNG} + \chi ^2$ method performed better compared to other methods.

**Fig. 9:**
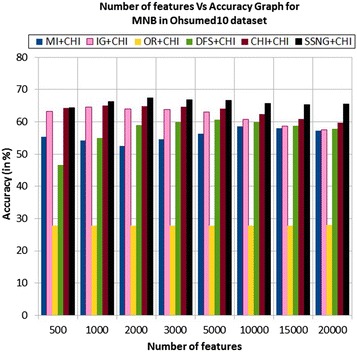
MNB on Ohsumed10 dataset

**Fig. 10:**
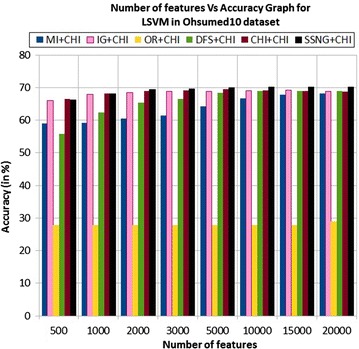
LSVM on Ohsumed10 dataset

In the Ohsumed10 dataset, the accuracy of the MNB classifier depends upon the number of features and achie-ves the peak value 67.32 % for 2000 numbers of features (see Table 7) the decreases and remain constant (see Fig. 9). In case of LSVM, the SSNG gains highest 70.18 % accuracy for 15,000 numbers of features (see Table 7) then decreases and remain constant (see Fig. 10). The success rate of SSNG method based on the TPF approach in Ohsumed10 dataset is 87.5 % because out of 16 experiments 14 times the $\hbox {SSNG} + \chi ^2$ method performed better compared to other methods.

**Fig. 11:**
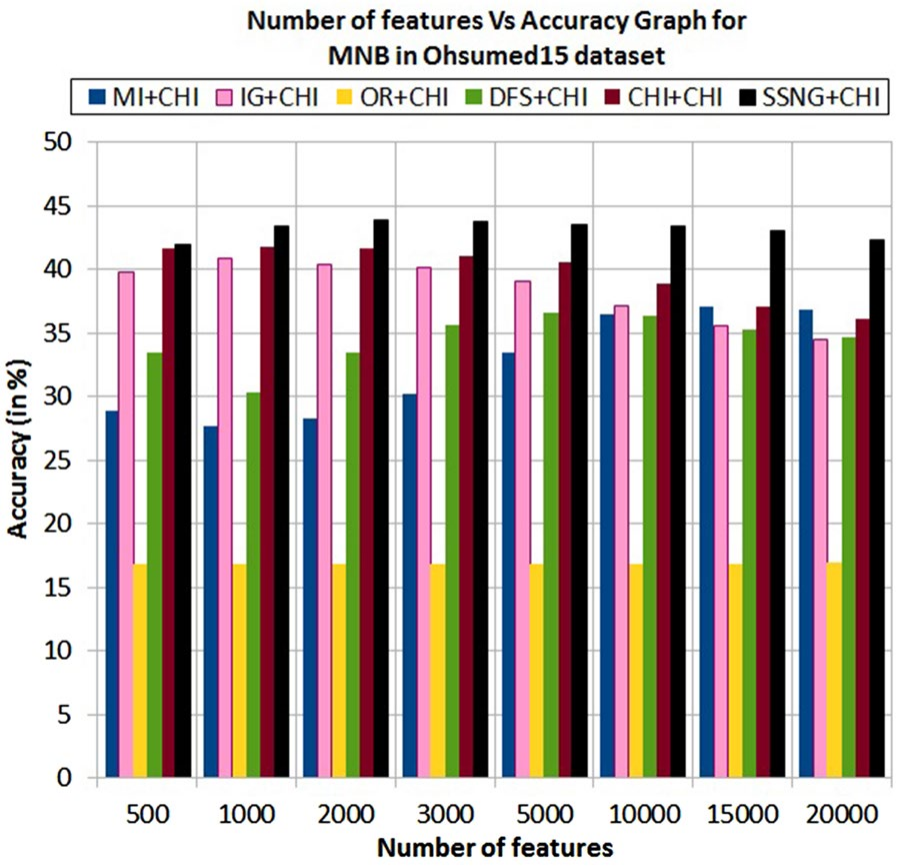
MNB on Ohsumed15 dataset

**Fig. 12:**
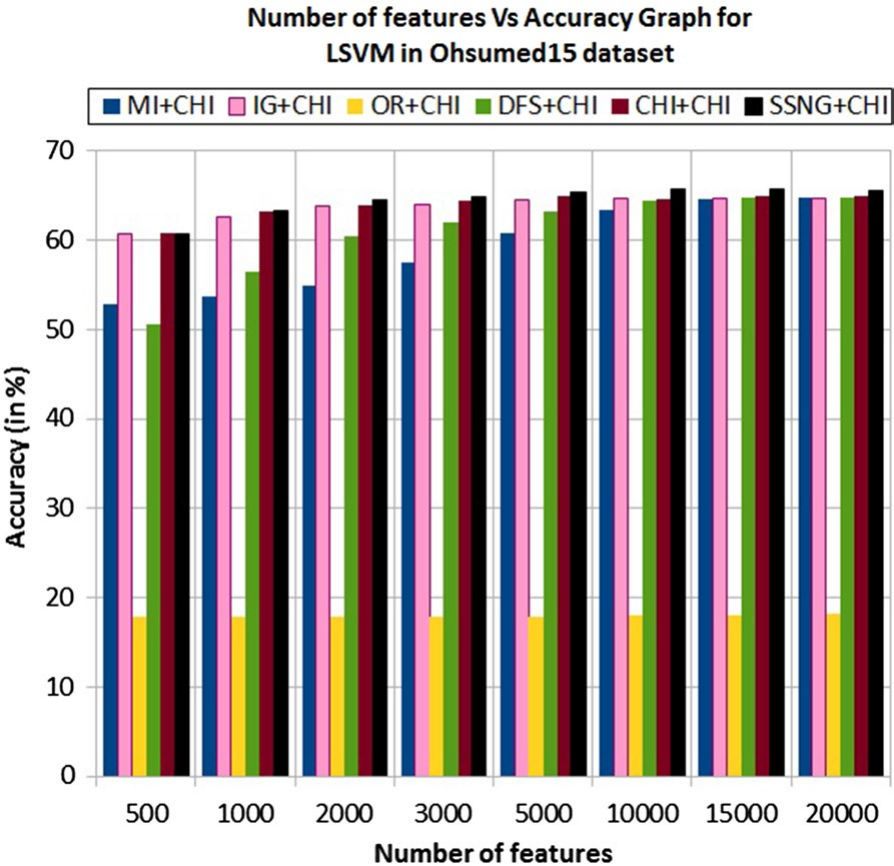
LSVM on Ohsumed15 dataset

In the Ohsumed15 dataset, the accuracy of the MNB classifier depends upon the number of features and achie-ves the peak value 43.91 % for 2000 numbers of features (see Table 7) then decreases and remain (see Fig. 11). In case of LSVM, the SSNG gains highest 65.75 % accuracy for 10,000 numbers of features (see Table 7) then decreases and remain constant (see Fig. 12). The success rate of SSNG in Ohsumed15 dataset is 93.75 % because out of 16 experiments 15 times the $\hbox {SSNG} + \chi ^2$ method performed better compared to other methods.

**Fig. 13:**
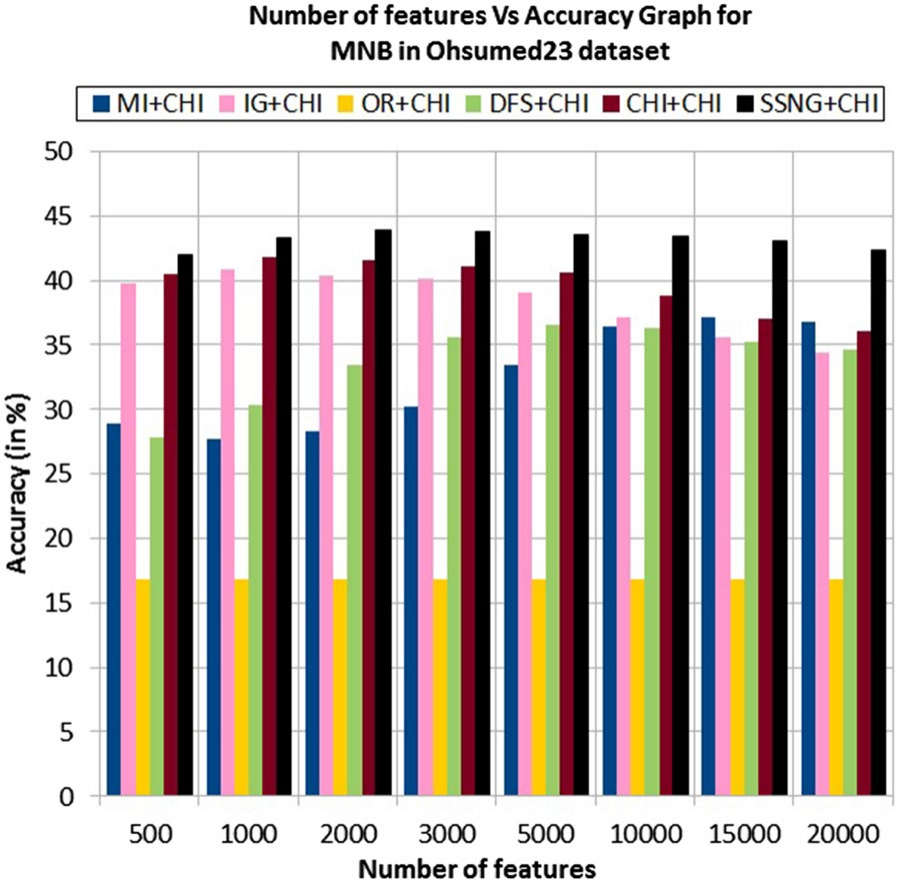
MNB on Ohsumed23 dataset

**Fig. 14:**
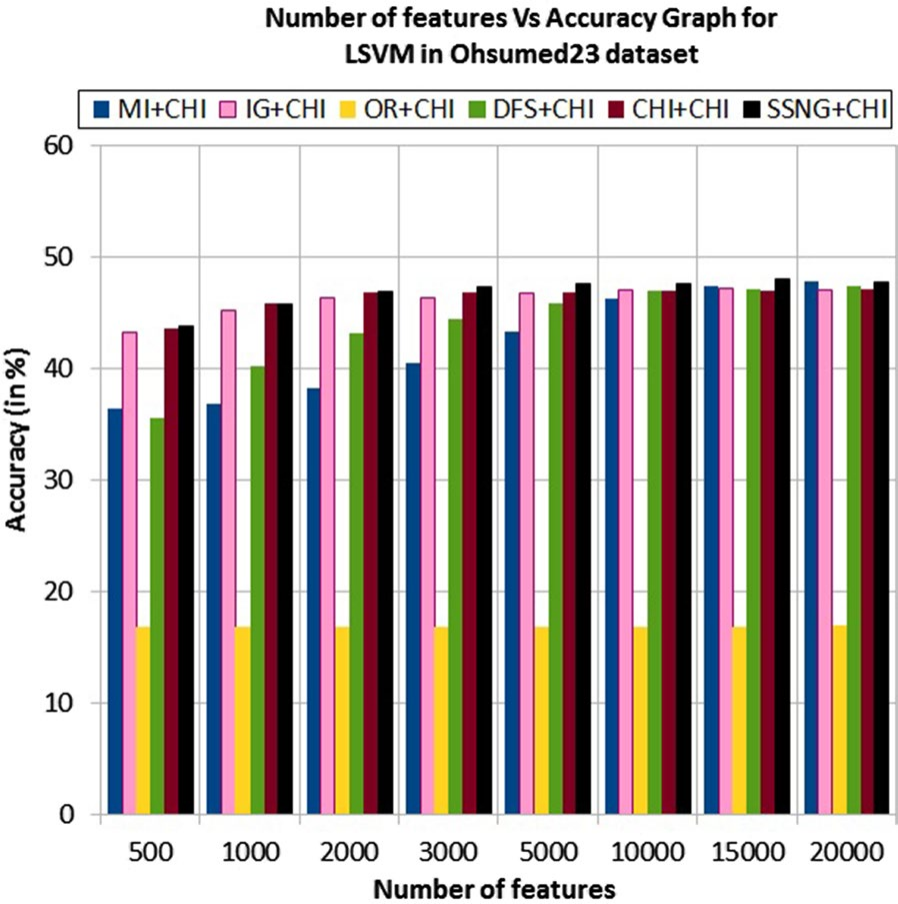
LSVM on Ohsumed23 dataset

In the Ohsumed23 dataset, the accuracy of the MNB classifier depends upon the number of features and achie-ves the peak value 43.91 % for 2000 numbers of features (see Table 7) then decreases and remain constant (see Fig. 13). In case of LSVM, the SSNG gains highest 48 % accuracy for 15,000 numbers of features (see Table 7) then decreases and remain constant (see Fig. 14). The success rate of SSNG in Ohsumed23 dataset is 93.75 % because out of 16 experiments 15 times the $\hbox {SSNG} + \chi ^2$ method performed better compared to other methods.

**Fig. 15:**
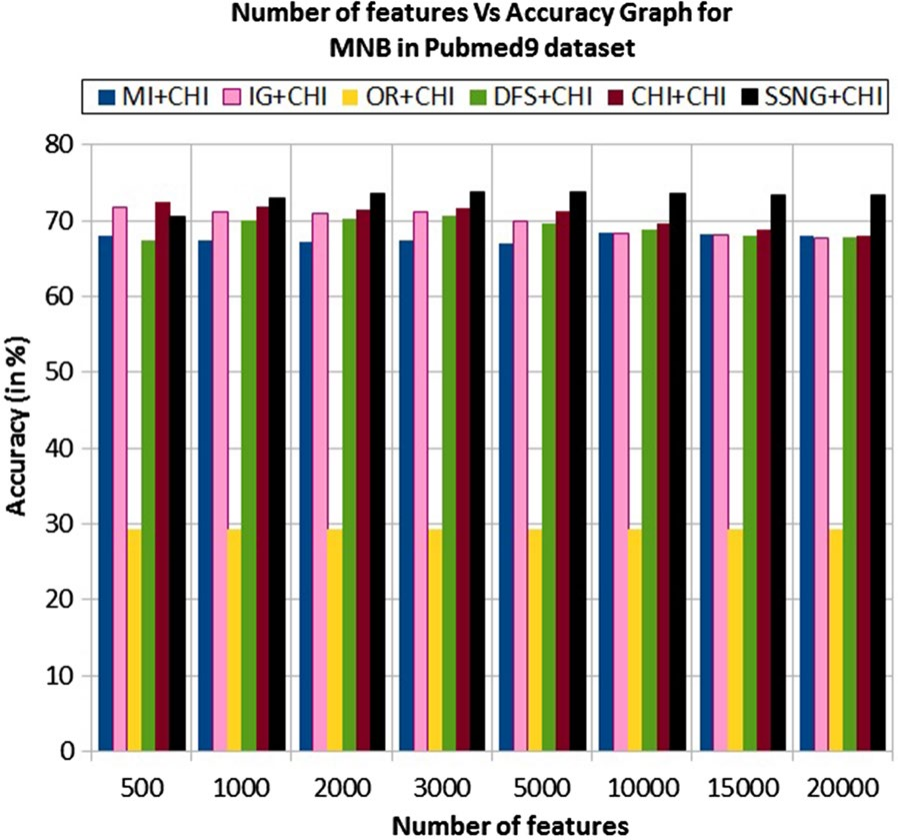
MNB on Pubmed9 dataset

**Fig. 16:**
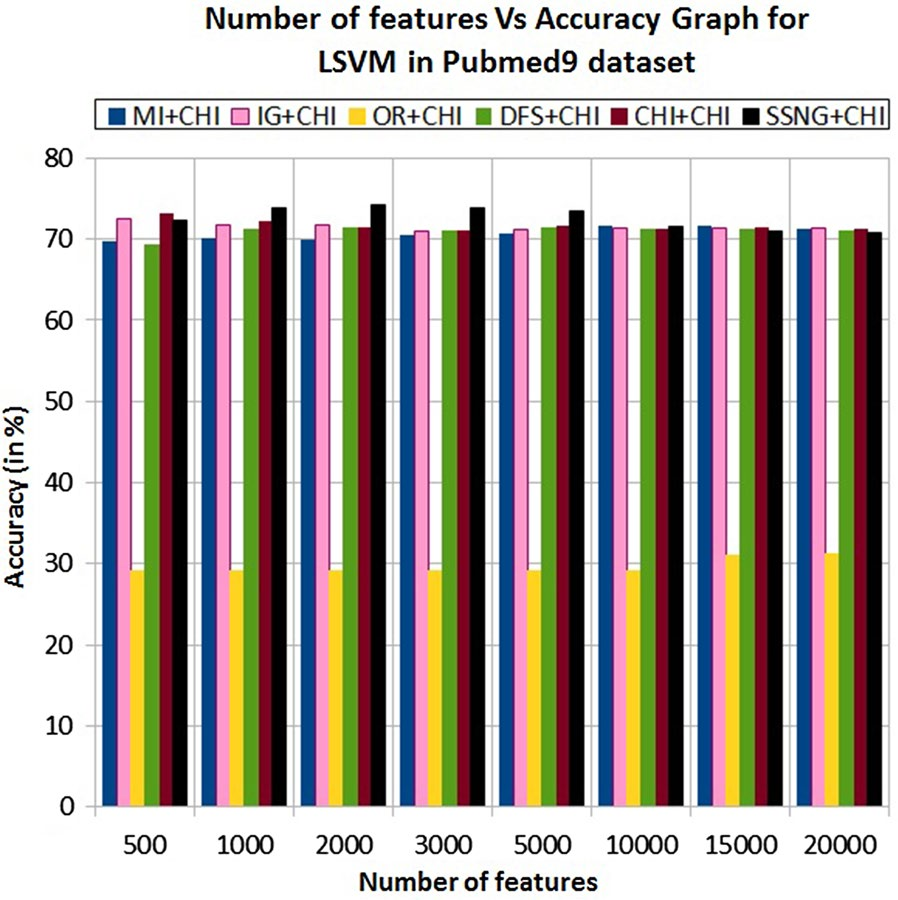
LSVM on Pubmed9 dataset

In the Pubmed9 dataset, the accuracy of the MNB classifier depends upon the number of features and achie-ves the peak value 73.84 % for 5000 numbers of features (see Table 7) then decreases and remain constant (see Fig. 15). In case of LSVM, the SSNG gains highest 74.15 % accuracy for 2000 numbers of features (see Table 7) then decreases and remain constant (see Fig. 16). The success rate of SSNG in Pubmed9 dataset is 68.75 % because out of 16 experiments 11 times the $\hbox {SSNG} + \chi ^2$ method performed better compared to other methods.

**Fig. 17:**
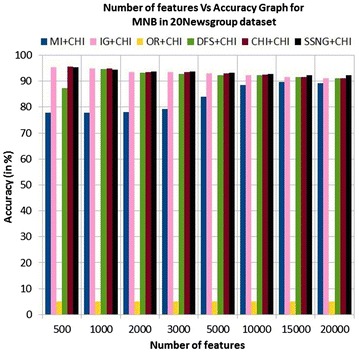
MNB on 20Newsgroup dataset

**Fig. 18:**
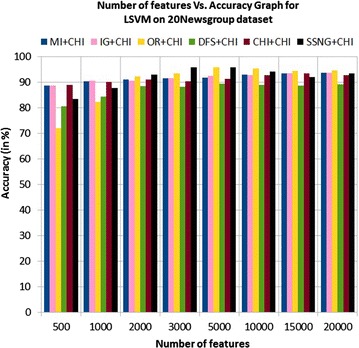
LSVM on 20Newsgroup dataset

In the 20Newsgroup dataset, the accuracy of the MNB classifier depends upon the number of features and achie-ves the peak value 95.6 % for 500 numbers of features (see Table 7) and then decreases and remain constant for features greater than 500 (see Fig. 17). In case of LSVM, the SSNG gains highest 95.8 % accuracy for 3000 and 5000 numbers of features (see Table 7) then decreases and remain constant (see Fig. 18). The success rate of SSNG method in 20Newsgroup dataset is 75 % because out of 16 experiments 12 times the $\hbox {SSNG} + \chi ^2$ method performed better compared to other methods.

**Fig. 19:**
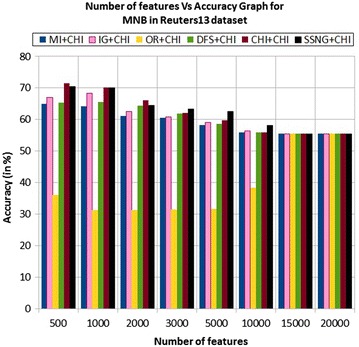
MNB on Reuters13 dataset

**Fig. 20:**
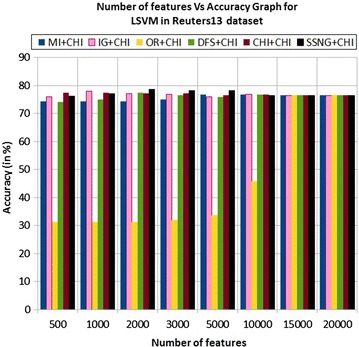
LSVM on Reuters13 dataset

In the Reuters13 dataset, the accuracy of the MNB classifier depends upon the number of features and achie-ves the peak value 71.59 % for 500 numbers of features (see Table 7) then decreases and remain constant (see Fig. 19). In case of LSVM, the SSNG gains highest 78.52 % accuracy for 2000 numbers of features (see Table 7) then decreases and remain constant (see Fig. 20). The success rate of SSNG in Reuters13 dataset is 62.5 % because out of 16 experiments 10 times the $\hbox {SSNG} + \chi ^2$ method performed better compared to other methods.

**Fig. 21:**
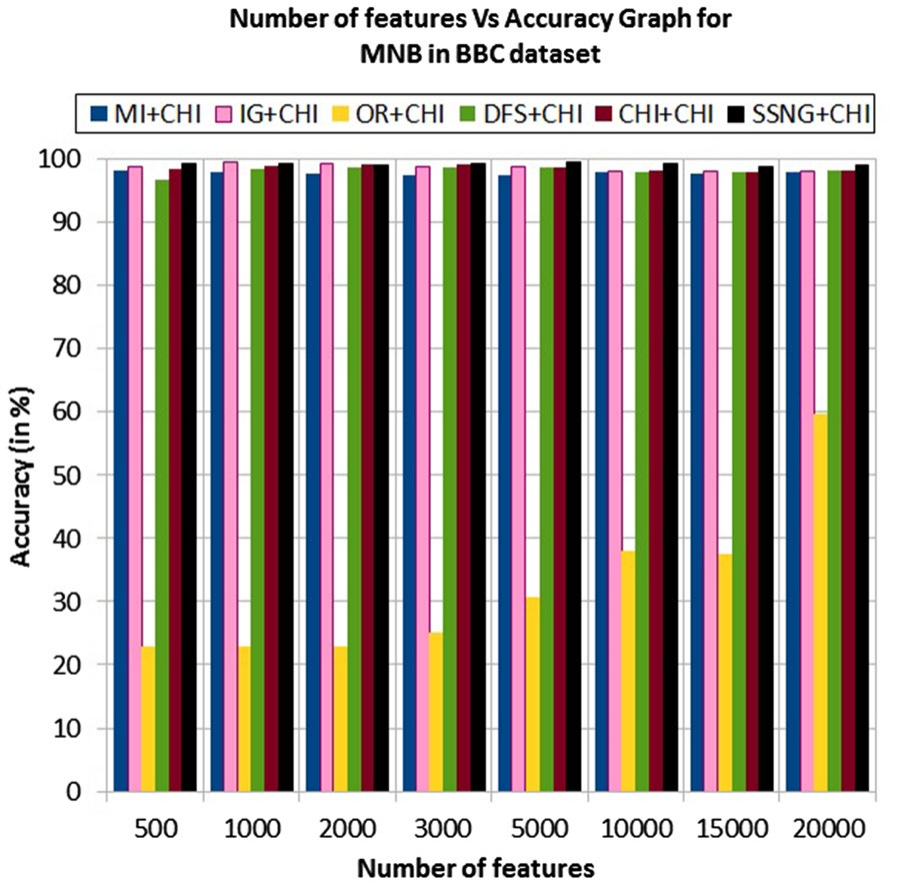
MNB on BBC news dataset

**Fig. 22:**
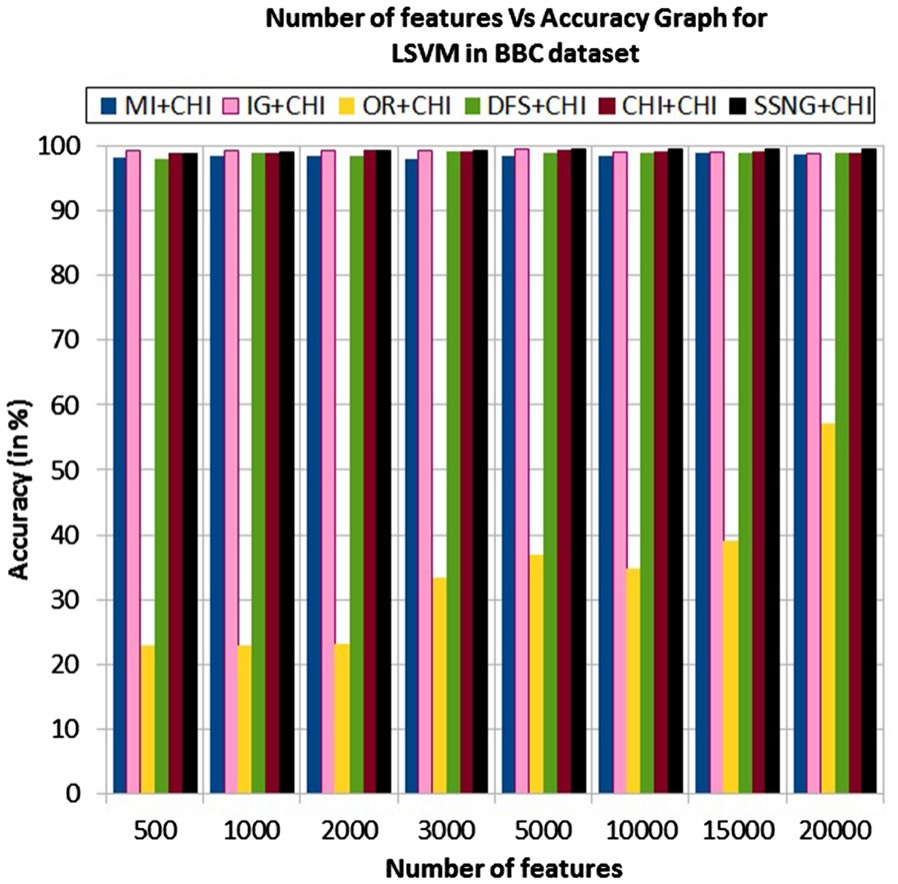
LSVM on BBC news dataset

In the BBC dataset, the accuracy of the MNB classifier depends upon the number of features and achie-ves the peak value 99.28 % for 1000, and 5000 numbers of features (see Table 7) then decrease and remain constant (see Fig. 21). In case of LSVM, the SSNG gains highest 99.64 % accuracy for 10,000 numbers of features (see Table 7) then decreases and remain constant (see Fig. 22). The success rate of SSNG in BBC dataset is 68.75 % because out of 16 experiments 11 times the $\hbox {SSNG} + \chi ^2$ method performed better compared to other methods.

**Fig. 23:**
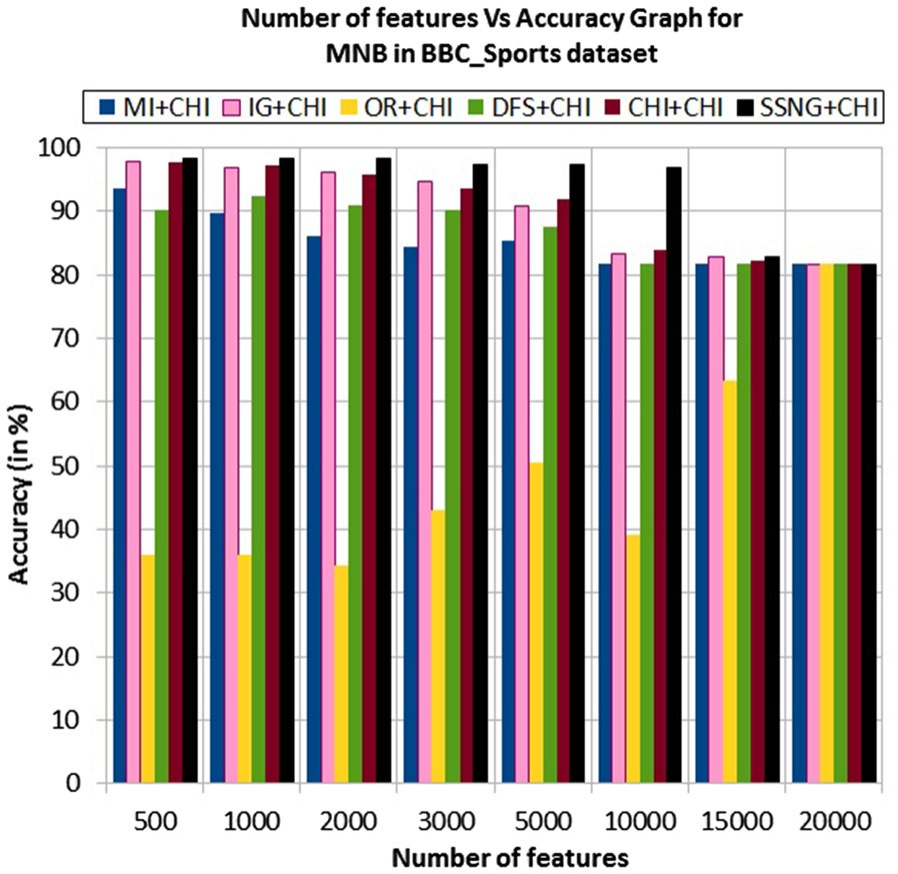
MNB on BBC_Sports news dataset

**Fig. 24:**
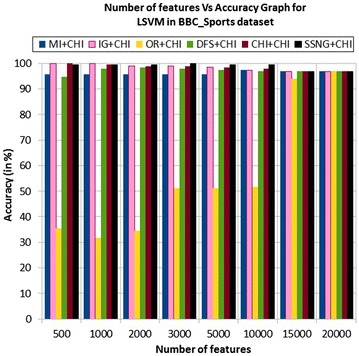
LSVM on BBC_Sports news dataset

In the BBC_Sports dataset, the accuracy of the MNB classifier depends upon the number of features and achie-ves the peak value 98.39 % for 500, 1000, and 2000 numbers of features (see Table 7) then decreases and remain constant (see Fig. 23). In case of LSVM, the SSNG gains highest 100 % accuracy for 500, 1000, and 3000 numbers of features (see Table 7) then decreases and remain constant (see Fig. 24). The success rate of SSNG in BBC_Sports dataset is 87.5 % because out of 16 experiments 14 times the $\hbox {SSNG} + \chi ^2$ method performed better compared to other methods.

In the experimental study, we have observed that

1. The accuracy of the classifiers have been found optimal, if the power $(NG_{SU} + NG_{Mem} + NG_{Strength})$ was selected as three and four of $NG_{RCST}$
2. It can be observed from Table 7, the proposed TPF based $\hbox {SSNG} + \chi ^2$ has given highest accuracy in nine datasets movie review, ACL IMDB, Ohsumed5, Ohsumed10, Ohsumed15, Ohsumed23, Pubmed9, BBC, and BBC_Sports, while in other two datasets 20Newsgroup and Reuters13, $\chi ^2+\chi ^2$ has given highest accuracy using MNB.
3. The success rate of the SSNG is 56.25 % for movie review, 68.75 % for ACL IMDB, 93.75 % for Ohsumed5, 87.5 % for Ohsumed10, 93.75 % for Ohsumed15 & Ohsumed23, 68.75 % for Pubmed9, 75 % for 20Newsgroup, 62.5 % for Reuters13 datasets, 68.75 % for BBC, and 87.5 % for BBC_Sports dataset.

## Conclusion

In this paper, a new text feature selection method symmetrical strength of N-Grams (SSNG method) has been introduced. It has improved the performance of the classifiers by assigning highest weight to the most informative N-Grams, while least weight to the non-informative N-Grams.

The SSNG has computed the weight of the N-Grams based on four probabilistic criteria- the symmetrical uncertainty, membership, strength, and the nature of the N-Grams. Further, the two pass filtering (TPF) based feature selection approach has been used to reduce the high dimensionality of the text data. In addition, we have discussed the problem related to representation of the terms using a well known BOW model. We followed the NGL model to generate the N-Grams to solve this problem. Initially, it has extracted more number of features due to NGL model, however, it is essential, to achieve high performance in terms of accuracy and f1_measure. The Apriori algorithm has been applied for pruning of the non-informative N-Grams.

The time complexity of the proposed TPF based SSNG method is higher than single filtered approaches, but the performance in terms of accuracy and f1_measure is more significant than single filtering approaches. The experimental study state the superior performance of the SSNG for the multi-class datasets, as well as two classes.

## Footnotes

1 http://www.isical.ac.in/~acmsc/TMW2014/TMW2014.html.

2 http://www.isical.ac.in/~scc/DInK%2710/studymaterial/textmining.

3 http://www.isical.ac.in/~acmsc/TMW2014/P_mitra.

4 https://en.wikipedia.org/wiki/Mutual_information.

5 http://www.nltk.org/%24nltk%5fdata%24/.

6 http://www.cs.cornell.edu/People/pabo/movie-review-data/.

7 http://ai.stanford.edu/~amaas/data/sentiment/.

8 http://archive.ics.uci.edu/ml.

9 http://trec.nist.gov/data/t9_filtering.html.

10 http://disi.unitn.it/moschitti/corpora.htm.

11 http://www.ncbi.nlm.nih.gov/books/NBK25501/.

12 http://cran.r-project.org/web/packages/reutils/reutils.

13 http://mlg.ucd.ie/datasets/bbc.html.

14 http://nbviewer.ipython.org/gist/rjweiss/7158866.

15 http://www.ncbi.nlm.nih.gov/books/NBK25501/.
